# Soil aggregate mediates the impacts of land uses on organic carbon, total nitrogen, and microbial activity in a Karst ecosystem

**DOI:** 10.1038/srep41402

**Published:** 2017-02-17

**Authors:** Shuangshuang Xiao, Wei Zhang, Yingying Ye, Jie Zhao, Kelin Wang

**Affiliations:** 1 Key Laboratory of Agro-ecological Processes in Subtropical Region, Institute of Subtropical Agriculture, Chinese Academy of Sciences, Changsha, Hunan 410125, China; 2 Huanjiang Observation and Research Station for Karst Ecosystems, Huanjiang, Guangxi 547100, China; 3 University of Chinese Academy of Science, Beijing 100039, China

## Abstract

Understanding the effect of land use on soil carbon, nitrogen, and microbial activity associated with aggregates is critical for thorough comprehension of the C and N dynamics of karst landscapes/ecosystems. We monitored soil organic carbon (SOC), total nitrogen (TN), microbial biomass carbon (MBC), and C_mic_: C_org_ ratio in large macro- (>2 mm), small macro- (0.25–2 mm), and micro- (0.053–0.25 mm) aggregates to determine the changes in soil properties under different land uses in the karst area of Southwest China. Five common land-use types—enclosure land (natural system, control), prescribed-burning land, fuel-wood shrubland, pasture and maize fields—were selected. Results showed that pasture and maize fields remarkably decreased the SOC and TN concentrations in aggregates. Conversion of natural system to other land uses decreased MBC (except for prescribed-burning) and increased C_mic_: C_org_ ratios in aggregates. The extent of the response to land uses of SOC and TN concentrations was similar whereas that of MBC and C_mic_: C_org_ ratios differed across the three aggregate sizes. Further, the SOC concentrations were significantly higher in macro-aggregates than micro-aggregates; the MBC and C_mic_: C_org_ ratios were highest in small macro-aggregates. Therefore, small macro-aggregates might have more active C dynamics.

Land-cover changes derived from land uses represent a major source and component of global environment change^1^,^2^. Intensive land use is the most significant anthropogenic activity and causes adverse effects on soil quality, such as soil structure destruction, nutrient loss, and soil erosion^3^,^4^,^5^,^6^. Moreover, land use changes can reduce soil C and N sequestration, resulting in the acceleration of greenhouse gas (CO_2_ and N_2_O) emissions^7^,^8^,^9^. Land-use changes also contribute to changes in microbial activities and biological processes that affect soil quality^10^,^11^.

The characteristics of vegetation and soil properties under different land uses have been extensively studied. For instance, deforestation not only affects forest structure, species composition and diversity^12^,^13^, but also reduces aboveground vegetation and litter return which directly affect soil nutrient cycling^14^,^15^. Fire can bring various impact on soil properties depending on the intensity and severity^16^,^17^,^18^. High-intensity fires such as many wildfires result in degradation of soil structure, loss of organic matter and microbes^17^. However, prescribed burning may minimize such negative effects on soil properties^18^. During management of pasture and cropland, aboveground plant biomass harvest and soil disturbance (sowing, weeding and tillage etc.) also influence soil structure and reduce soil nutrient and microbial biomass^19^,^20^.

Soil aggregates, the basic units of soil structure, are sensitive to land uses and mediate many chemical and biological processes in soils^19^,^21^,^22^,^23^,^24^. According to the hierarchical model proposed by Tisdall and Oades^25^, free primary particles and silt-sized aggregates are bound together into micro-aggregates by persistent binding agents, oxides, and highly disordered aluminosilicates. Furthermore, these stable micro-aggregates form macro-aggregates via temporary and transient binding agents (i.e. fungal hyphae and roots). The hierarchical order of aggregates might lead to the differences in the distribution and availability of soil organic matter (SOM)^22^,^26^. Previous studies have shown that land use can affect the C and N within aggregates, and the degree of its influence depends on soil texture and management measures^3^,^4^,^27^. Hence, investigating the dynamics of soil C and N associated with aggregates in various land uses is essential for thoroughly understanding the nutrient cycling process.

Soil microorganisms associated with aggregates play a key role in C and N dynamics following cultivation or other disturbances^23^. Each type of aggregate represents a different ecological niche for microbes because of the spatial arrangement of solid particles and accessible substrates within fraction^22^,^28^. The microbial biomass in soil aggregates has been shown to be heterogeneously distributed^9^,^29^,^30^; however, results have been inconsistent across studies. Furthermore, the C_mic_: C_org_ ratio, a microbial parameter, indicates C_org_ available for microbial growth^31^. Recent studies have shown that aggregate size determines the utilization of available C by microorganisms^22^,^30^. Further, the degree of the effects of soil disturbance on microbial activity within aggregates was shown to be mediated by fraction size^22^. However, information on how land uses affect microbial activities associated with aggregates in fragile karst ecosystems is limited.

Karst areas account for about 12% of the Earth’s land. In China, karst landscapes are mainly distributed in the southwest regions, which are subjected to extreme environmental conditions such as rapid organic matter loss, soil degradation, and rocky desertification^32^. Previous studies on karst regions have mainly focused on the C, N, and microbe levels in bulk soil, but not in aggregates^33^,^34^. Hence, how land uses affect the C, N, and microbial activity associated with aggregates in karst areas remains unclear. Understanding the influence of land uses on soil processes is critical for deciding land management strategies in the karst regions. Therefore, we chose five typical land uses (enclosure land, prescribed-burning land, fuel-wood shrubland, pasture and maize fields) in karst regions, and aimed to identify the effects of various land uses on the SOC, TN, and microbial activity (MBC and C_mic_: C_org_ ratio) associated with aggregates. Considering different land uses had various vegetation management and soil disturbance regimes, we hypothesized that (1) conversion of the natural system to other land uses would decrease aggregate-associated SOC, TN, and microbial activity, and the extent of land-use effects would differ across the three sizes of aggregates; (2) SOC and TN concentrations and microbial activity would be higher in macro-aggregates as indicated by the aggregate hierarchical model; (3) different land uses would affect SOC, TN and microbial activity via various directly or indirectly pathways.

## Results

### Size distribution of water-stable aggregates

Large macro-aggregates represented the greatest fraction for all land uses, whereas micro-aggregates represented the lowest proportion (Table 1). The level of large macro-aggregates was significantly lower in the prescribed-burning land (54.95%) and maize field (53.94%) than in the enclosure land (72.77%). Small macro-aggregates were significantly higher in the prescribed-burning land, pasture, and maize field (32.38%, 22.65%, and 30.29%, respectively). Compared with enclosure land, other four land uses had no significantly difference in micro-aggregates (*P* > 0.05).

### SOC and TN concentrations within soil aggregates

**Figure 1:**
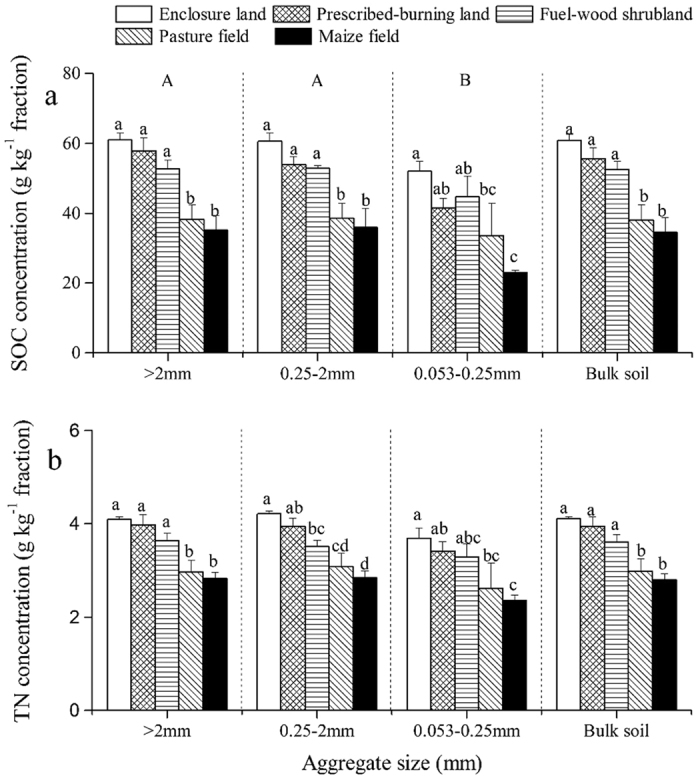
Soil organic carbon (SOC) (**a**) and total nitrogen (TN) (**b**) concentrations in the three sizes of soil aggregates and in bulk soil of different land uses. Values are means of three replicates (± standard error). Different lowercase letters indicate significant differences among land uses for each size of soil aggregate and for bulk soil. Different uppercase letters indicate significant differences among the three sizes of soil aggregates.

According to the two-way ANOVA, land uses and soil aggregate sizes significantly affected SOC and TN concentrations within soil aggregates (Table 2). Enclosure land had the highest SOC and TN concentrations in the three sizes of aggregates and bulk soil, followed by prescribed-burning land and fuel-wood shrubland, and pasture and maize field had relatively lower SOC and TN concentrations (Fig. 1). However, the SOC and TN concentrations in prescribed-burning land and fuel-wood shrubland were not significantly different from those in the enclosure land (*P* > 0.05); their concentrations were significantly lower in pasture and maize fields. Further, the SOC concentration in micro-aggregates was remarkably lower than that in macro-aggregates.

### SOC and TN stocks within soil aggregates

**Figure 2:**
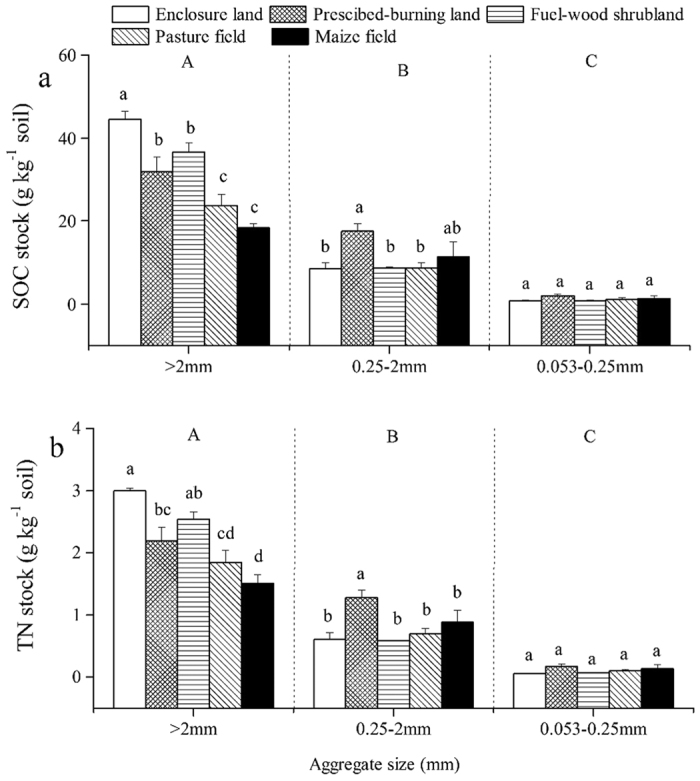
Soil organic carbon (SOC) (**a**) and total nitrogen (TN) (**b**) stocks in the three sizes of soil aggregates and in bulk soil of different land uses. Values are means of three replicates (± standard error). Different lowercase letters indicate significant differences among land uses for each size of soil aggregate and for bulk soil. Different uppercase letters indicate significant differences among the three sizes of soil aggregates.

Land uses and soil aggregate sizes significantly affected SOC and TN stocks within soil aggregates (Table 2). And the interaction between land uses and aggregate sizes was also remarkable. Large macro-aggregates comprised the major SOC and TN pools regardless of the land uses (Fig. 2). They contained 18.35–44.59 g C · kg^−1^ soil and 1.51–2.99 g N · kg^−1^ soil, accounting for 58.76–82.54% of SOC and 59.68–81.92% of TN. Further, the micro-aggregates had the lowest SOC and TN pool regardless of land uses. In general, our results showed that SOC and TN stocks in large macro-aggregates significantly decreased when the natural vegetation was converted to other land uses. In small macro-aggregates, the SOC and TN stocks in prescribed-burning land were higher by 8.96 g · kg^−1^ and 0.68 g · kg^−1^ than those in the enclosure land, respectively. The SOC and TN stocks in micro-aggregates were not remarkably different across the land uses (*P* > 0.05).

### Microbial biomass C within soil aggregates

**Figure 3:**
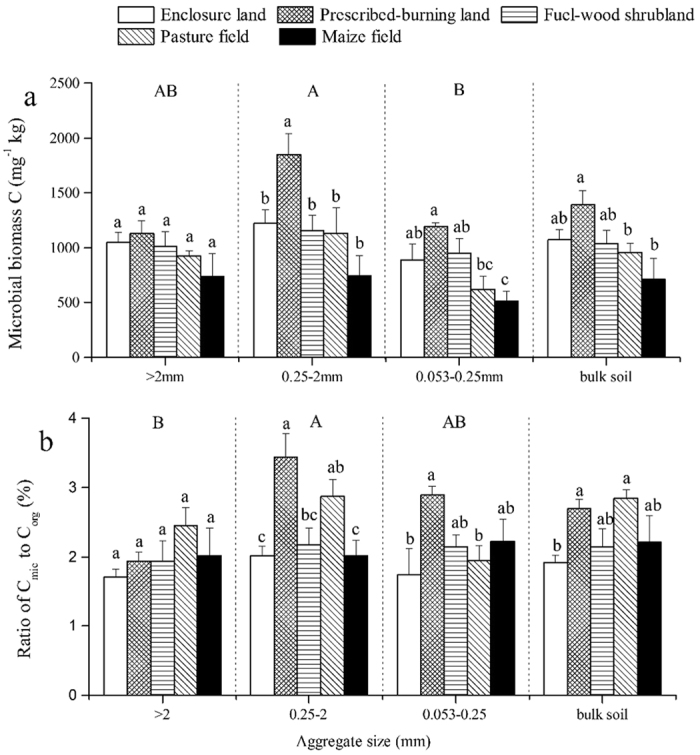
Microbial biomass carbon (MBC) (**a**) and the C_mic_: C_org_ ratios (**b**) of the three sizes of soil aggregates and bulk soil of different land uses. Values are means of three replicates (± standard error). Different lowercase letters indicate significant differences among land uses for each size of soil aggregate and for bulk soil. Different uppercase letters indicate significant differences among the three sizes of soil aggregates.

Land uses and soil aggregate sizes significantly affected MBC within soil aggregates, respectively (Table 2). MBC in aggregates and bulk soil in other land uses (except for prescribed-burning) decreased compared with that in enclosure land (Fig. 3a). Further, the maize field had the lowest MBC. Moreover, the MBC in small micro-aggregates of prescribed-burning land (1850.62 mg · kg^−1^) was significantly higher than that of enclosure land (1219.90 mg · kg^−1^). The pasture and maize fields had much lower MBC in micro-aggregates (623.36 mg · kg^−1^ and 514.30 mg · kg^−1^, respectively) than in prescribed-burning land (1191.05 mg · kg^−1^). However, the MBC in large macro-aggregates did not differ significantly among all land uses. In the three aggregates, MBC was the highest in small macro-aggregates, followed by large macro-aggregates and micro-aggregates.

### C_mic_: C_org_ ratios within aggregates

Land uses and soil aggregate sizes significantly affected the C_mic_: C_org_ ratios within soil aggregates, respectively (Table 2). The C_mic_: C_org_ ratios ranged between 1.71% and 3.44% in our study (Fig. 3b). Compared to enclosure land, the ratios in other land uses increased in aggregates and bulk soil. The highest C_mic_: C_org_ ratio (3.44%) was observed in small macro-aggregates from prescribed-burning land; this ratio was significantly higher than that of enclosure, fuel-wood shrubland, and maize field (2.01%, 2.17%, and 2.01%, respectively). However, no significant differences in the C_mic_: C_org_ ratios in large macro-aggregates were identified for all land uses (*P* > 0.05). Among the three aggregates, small macro-aggregates had the highest C_mic_: C_org_ ratios, followed by micro-aggregates; large macro-aggregates had the lowest ratio.

### Principal components analysis

Two principal components (PCs) with eigenvalues of >1 were extracted from the soil water-stable aggregates (WSAs), soil nutrient status (SOC, TN), and microbial activity (Table 3). The highest component loadings of the first PC (49.55% of total variance) included water-stable aggregates that indicated the soil physical structure. The second PC (36.78% of total variance) was related to SOC, TN, and MBC, which reflected soil biochemical variables. ANOVA results showed that the first PC of enclosure land differed from that of prescribed-burning land and maize field (*P* < 0.05), indicating that these two land uses could change soil physical structure more easily. Further, the second PC of pasture and maize fields was remarkably different from that of enclosure land, indicating that forage and crop cultivation had a marked impact on soil biochemical properties.

## Discussion

Soil aggregation is commonly facilitated by vegetation restoration caused by SOC return^35^. Our study showed that enclosure land facilitated the formation of large macro-aggregates (>2 mm). Pasture field also had a comparatively high level of large macro-aggregates because of the strong plant root systems that are beneficial for soil aggregation^36^. Prescribed-burning land showed a lower amount of large macro-aggregates, probably because of the extreme heat that destroyed soil aggregates. Intra-aggregate water is vaporized when burning takes place, and the increased pressure causes the internal bonds to rupture, leading to aggregate breakdown^24^. Moreover, maize field had the lowest amount of large macro-aggregates. Physical disturbance resulting from tillage might be responsible for breakage of the large aggregates^19^.

Our study showed that macro-aggregates (>0.25 mm) were dominant in water-stable aggregates in all land uses, which is consistent with the results of Liao *et al*.^37^, who also conducted a study in the karst region. In addition, macro-aggregate quantity was markedly influenced by land uses, whereas micro-aggregates were more stable than macro-aggregates (Table 1). Previous studies have shown that macro-aggregates are vulnerable to soil disturbance because their transient and temporary binding agents such as roots and mycelia are sensitive to disturbance^3^,^38^.

Consistent with our first hypothesis, conversion of enclosures to other land uses deceased SOC and TN concentrations in aggregates and bulk soil (Fig. 1, Table 2). However, aggregate-associated SOC and TN concentrations did not decrease significantly in prescribed-burning land and fuel-wood shrubland (*P* > 0.05). With regard to prescribed-burning land, the lack of a significant change may be due to conversion of aboveground biomass C to surface soil C by fire^18^. Another explanation is that pyrogenic carbon produced from combustion is resistant to degradation^39^. SOC and TN concentrations associated with aggregates in agricultural land uses (pasture and maize fields) were remarkably reduced, which was consistent with the results of Udom and Ogunwole^40^. In agricultural ecosystems, decreases in SOC are mainly induced by frequent soil disturbance (e.g. tillage, fertilization, and weed control) and crop removal^20^.

In contrast to the predictions of our hypothesis, the magnitude of land-use effects on SOC and TN concentrations was the same for all three sizes of aggregates. However, other studies have reported that these extents were less pronounced in micro-aggregates than in macro-aggregates^5^. They considered that SOM in micro-aggregates was more stable owing to the persistent binding agents (oxides and highly disordered aluminosilicates). However, calcareous soil in the karst region has high contents of calcium and clay, and SOM in the micro-aggregates might be immobilized by Ca^2^ (acting as a cationic bridge)^41^. Disturbance-induced soil Ca^2+^ and clay loss might be accompanied by SOM decrease in the karst area^32^. This might explain why the magnitude of land-use impact on SOC and TN concentrations in micro-aggregates was the same as that in macro-aggregates. In addition, the SOC concentration was significantly higher in macro-aggregates than in micro-aggregates (Fig. 1, Table 2), which was consistent with our second hypothesis and similar with the findings of previous studies^40^,^41^,^42^. Further, this is in accordance with the hierarchical model that macro-aggregates consist of micro-aggregates and transient and temporary organic binding agents (i.e. fungal hyphae and roots)^25^.

Our results showed that large macro-aggregates (>2 mm) dominated the SOC and TN storage of aggregates in all land uses; this was caused by the large number of macro-aggregates rather than the SOC and TN concentrations. Moreover, the SOC and TN stocks in large macro-aggregates were significantly reduced after the conversion of natural system to other land uses. The SOC and TN stocks in smaller fraction sizes were not significantly different across all land uses (except for prescribed-burning). The results indicated that the SOC and TN of bulk soil were primarily lost by the decreases of SOC and TN stocks in large macro-aggregates. The significant reduction of SOM stock in large macro-aggregates in the maize field was attributed to both the aggregate fraction (>2 mm) quantity and SOM concentrations within the fraction (Table 1, Fig. 1), which was consistent with the results of Qiu *et al*.^27^. Periodic fires caused marked alterations in SOC and TN stocks from large to small macro-aggregates owing to the destruction of large aggregates.

Soil MBC generally decreases when natural systems are disturbed or converted to other land uses^2^,^43^. This is consistent with our results that MBC in aggregates and bulk soil decreased in other land uses (except for prescribed-burning; Fig. 3a), because natural vegetation contains plant litters that supply C inputs and reduces water and heat exchange^2^. Moreover, natural systems have good physical structure without soil disturbance. These factors contribute to the maintenance of a suitable microenvironment for microbial population. However, our result showed prescribed-burning increased the MBC, which was inconsistent with results of a meta-analysis^44^. One possible explanation for this is that soil samples were collected only a few days after prescribed fire in our study. The NH_4_^+^-N concentration and total microbial biomass might increase in a short time after burning^45^. Low C_mic_: C_org_ ratio in soil under intensive cultivation is an indicator of soil degradation after conversion from natural to agricultural systems^2^,^46^. However, our results showed that enclosure land had the lowest C_mic_: C_org_ ratio in aggregates and bulk soil, and that prescribed-burning and pasture fields had relatively high C_mic_: C_org_ level (Fig. 3b). However, enclosure land had many large macro-aggregates that caused more SOC and relatively lower microbes. Planting crops decreased both the MBC and SOC, but the reduction of SOC was more than that of MBC that led to the high C_mic_: C_org_. The elevated MBC after prescribed burning was the main cause for the increase in C_mic_: C_org_.

Land uses have great impacts on microbial activity (MBC and C_mic_: C_org_ ratio) in small macro-aggregates and micro-aggregates, but not in large macro-aggregates (Fig. 3b). This is mainly because the large radius of large aggregates could limit the O_2_ concentration and gas diffusion required by microbes^23^,^30^. Thus, large macro-aggregates might diminish the impacts of land uses and facilitate the maintenance of a stable microbial biomass. In addition, the highest MBC was found in small macro-aggregates (0.25–2 mm) regardless of land uses (Fig. 3a). Previous studies have shown that soil aggregate size exerts strong impacts on microbial activity, and that microbial biomass is heterogeneously distributed among aggregates^29^,^30^,^47^. A similar result was obtained by Jiang *et al*.^30^, who found that the highest MBC appeared in 1–2 mm fractions. Two possible explanations can be suggested: first, small macro-aggregates have more SOC and TN for microbial growth than micro-aggregates (Fig. 1); second, soil microorganisms live in aggregate pores or at their surfaces, and small macro-aggregates have a more suitable radius and higher surface area than large macro-aggregates^48^. Moreover, our results showed that large macro-aggregates had significantly lower C_mic_: C_org_ ratio than small macro-aggregates (Fig. 3b). The C_mic_: C_org_ ratio indicated that C_org_ was available for microbial growth^31^. Our results suggested that large aggregates limited SOC availability by microbes, and medium size of aggregates was the most suitable for SOC utilization.

**Figure 4:**
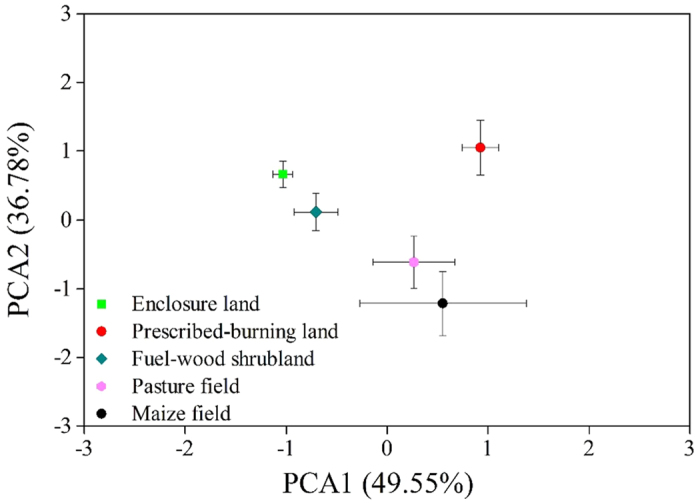
Principal component analysis (PCA) of water-stable aggregates, soil organic carbon, total nitrogen, and microbial activity in the five land use types. Error bars represent the standard error of the means.

Taken together, our findings suggested that five land uses affect soil physical and biochemical properties to different degrees through various pathways (Table 3 and Fig. 4). Without soil disturbance, the fuel-wood land had no significant change in soil physical structure compared to enclosure. And fuel-wood harvest did not remarkable altered the soil biochemical properties, which might indicate aboveground vegetation removal in a low frequency was insufficient to induce significant changes of soil properties. The soil biochemical status of other three land uses was influenced both by vegetation variety and indirectly by aggregate composition alteration owing to soil disturbance. Prescribed-burning reduced the nutrient return from vegetation and brought about the fragmentation of aggregates. However, pyrogenic carbon formed by combustion decreased the loss of soil C and N owing to aggregate rupture^39^. Poor soil biochemistry of maize field was caused by both aboveground biomass harvest and soil physical disturbance derived from cultivation. Aggregate breakage due to physical perturbation may lead to the mineralization and loss of large amount of free organic carbon and nitrogen^19^. The reduction process of soil nutrient in pasture field was similar with maize. But because of strong root system, the pasture could maintain relatively good soil physical structure^36^.

## Conclusions

The amount of large macro-aggregates was significantly lower in prescribed-burning and maize field, and the effects of land uses on macro-aggregate quantity were stronger than those on micro-aggregate quantity. The conversion of natural systems to other land uses decreased the SOC and TN concentrations in aggregates and bulk soil. Maize and pasture fields showed significant reduction in these parameters, and their loss of soil C and N were mainly attributed to the reduction of SOC and TN stocks in the large macro-aggregates. The response degree of SOC and TN concentrations to land uses was similar across the three sizes of soil aggregates. As expected from the hierarchical model, SOC concentration was significantly higher in macro-aggregates than micro-aggregates.

The conversion of natural vegetation to other land uses decreased the MBC (except for prescribed-burning) and increased the C_mic_: C_org_ ratios in aggregates and bulk soil. The impact of land use on microbial activity was less pronounced in large macro-aggregates. In addition, the MBC and C_mic_: C_org_ ratios were the highest in small macro-aggregates, which might indicate that the medium fraction contained more available SOM and was better suited for microorganism growth. Hence, our results suggested that the conversion of natural vegetation to the four land uses reduced not only large macro-aggregate quantity but also SOC and TN concentrations and MBC in aggregates as well as in bulk soil. Additionally, the magnitude of the response of microbial activity to land uses was different across the three sizes of aggregates. Small macro-aggregates had higher SOC, TN concentrations, and microbial activity, suggesting more active C and N dynamics. These findings suggested that aggregate size should be explicitly considered to determine the impact of management practices on soil quality, and provided theoretical basis for deciding reasonable land use for conservation and ecological restoration in the karst region.

## Materials and Methods

### Site description and experimental design

**Figure 5:**
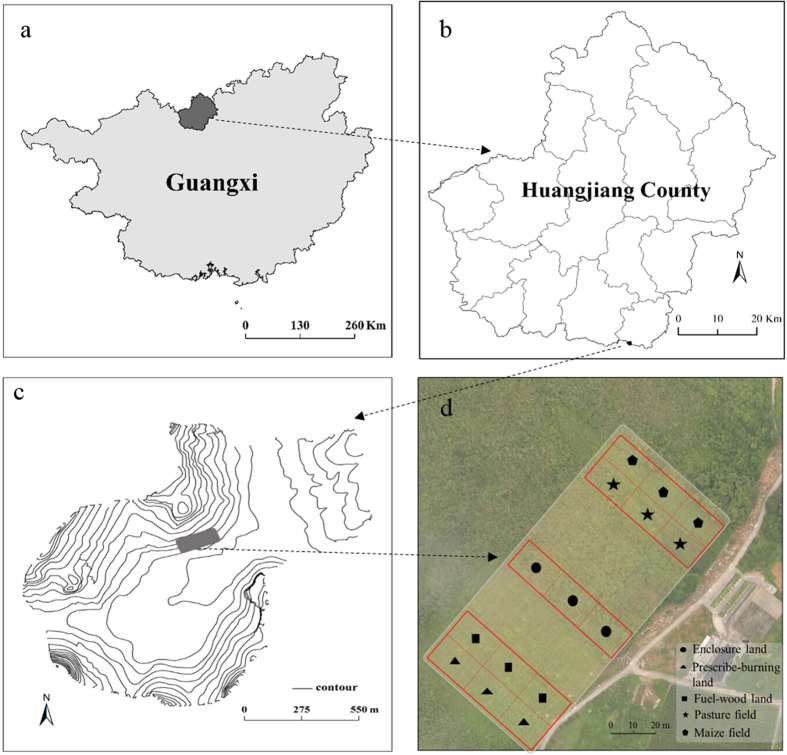
Map of region and field area. (**a**) Location map of Huanjiang County within the Guangxi Province, China. (**b**) Location map of the studied catchment within the Huangjiang County. (**c**) Location of long-term observation field. (**d**) The layout of sampled land use plots. (**a**–**c** maps were created by ArcGIS 9.3 URL: http://www.esrichina.com.cn/softwareproduct/ArcGIS/; (**d**) photo were taken by Wei Luo & Xianli Xu using aerial instrument DJI Phantom 3 URL: http://www.dji.com/cn/products/phantom-3. Here we appreciate their supply).

This study was performed at the Huanjiang Observation and Research Station for Karst Ecosystems (107°51′–108°43′E, 24°44′–25°33′N), Chinese Academy of Sciences (GAS), Guangxi Province, China (Fig. 5). The climate is subtropical monsoon with distinct wet (from April to September) and dry (from October to March) seasons. The mean annual temperature and precipitation are 18.5 °C and 1,380 mm, respectively^36^. The soil developed from a dolostone base and is calcareous^49^.

The experiment site was formerly used as cultivated land that was fallow after 1985, when the residents relocated. Through 21 years the natural vegetation had been restored, and the common plant species included *Sapium rotundifolium, Vitex negundo*, and *Artemisia hedinii*. At the end of 2006, adjacent and relatively homogeneous areas with similar topography and slope were selected, and five kinds of long-term observation fields (enclosure land, prescribed-burning land, fuel-wood shrubland, pasture, and maize field) were established. All the land use types have similar physiographic conditions and slope gradients. We therefore assume that the soils in the five land use types had similar initial conditions. Three replicated plots (20 m × 20 m) were established within each land use treatment. In the prescribed-burning land, burning was usually implemented once a year in December, and aboveground vegetation was completely burned off. The plants in fuel-wood shrubland were logged and removed without root disturbance in December. The pasture field was used to grow a perennial hybrid napier grass (*Pennisetum hybridum*). Basal fertilizer was applied annually after the pasture became green (45 kg N, 45 kg P_2_O_5_, and 45 kg K_2_O · hm^−2^). The pasture field was mowed every four months, but not ploughed. The maize field was fertilized with N, P, and K at 160, 90, and 90 kg · hm^−2^, respectively, in the growing period. As a control, enclosure lands retained intact vegetation without any soil disturbance. The soil properties are shown in Table 4.

### Soil sampling and aggregate fractionation

All surface soil (0–15 cm) samples were collected from each plot in January 2014. One undisturbed soil sample was taken in separate pits (15 cm height × 20 cm length × 20 cm width) after the residue was removed. According to “S” type, five samples were collected from each plot and mixed together to obtain one sample for the replicate. The microbes were preserved by storing the samples under cooled conditions (4 °C) before further preparation. Visible roots, organic residues, and stone fragments were removed manually. Soil samples were gently crushed and divided into two portions: one was passed through an 8-mm sieve for determining aggregate-size distribution and fractionation, and the other one was passed through a 2-mm sieve for the determining the soil bulk properties.

Soil water-stable aggregates (WSAs) were fractionated using a wet-sieving procedure^30^,^50^. Field-moist soil was immersed in water on a set of three nested sieves (2, 0.25, and 0.053 mm) and shaken vertically at 3 cm for 50 times for 2 min. The aggregates retained on each sieve were collected. The soil aggregates were separated as large macro-aggregates (>2 mm), small macro-aggregates (0.25–2 mm), and micro-aggregates (0.053–0.25)^9^,^21^. Bulk soil samples and separated aggregates were analysed for biochemical characteristics.

### Microbial and chemical analyses

SOC was measured by the Walkley-Black wet-chemical oxidation method^51^. TN was determined with an Element Auto-Analyser (Vario MAX CN; Elementar, Hanau, Germany). Stock calculated as SOC and TN concentration in an aggregate multiplied by the amount of the aggregate in one kilogram soil^42^. Soil microbial biomass C was determined by fumigation extraction^52^. Briefly, 10 g of fumigated and unfumigated soil samples were extracted with 0.5 M K_2_SO_4_ in 1:4 ratio. Since not all the microbial C was extracted by K_2_SO_4_, a *k* factor of 0.45 was used to convert microbial C flush to MBC.

### Statistical analysis

The contents of WSAs, SOC, TN, and microbial activity within aggregates were compared among the five land uses by using one-way analysis of variance (ANOVA) to test for differences. Fischer’s least significant difference post hoc test was used to separate significant differences among the land uses at *P* < 0.05 significance level. Two-way analysis of variance (ANOVA) was conducted to test the effects of land use and aggregate size on SOC, TN concentration and stocks, MBC and C_mic_: C_org_ ratio within soil aggregates. The least significant difference (LSD) between any two means was calculated using the Student’s *t* test at the 5 percent level. In addition, principal components analysis (PCA), which is often performed to eliminate multicolinearity and reduce the number of variables in a data set for accurate data analysis, was performed by considering most or all of the information. Water-stable aggregates (WSAs), soil nutrient status (SOC, TN), and microbial activity were analysed by transforming the data to their principal components and subjecting them to ANOVA^53^,^54^. PCAs and ANOVA were performed using SPSS 16 (SPSS Inc., Chicago, IL, USA).

## Additional Information

**How to cite this article**: Xiao, S. *et al*. Soil aggregate mediates the impacts of land uses on organic carbon, total nitrogen, and microbial activity in a Karst ecosystem. *Sci. Rep.* **7**, 41402; doi: 10.1038/srep41402 (2017).

**Publisher's note:** Springer Nature remains neutral with regard to jurisdictional claims in published maps and institutional affiliations.

## Figures and Tables

**Table 1:** Aggregate size distribution (%) of soils under different land uses.

Type	Aggregate size (mm)		
	>2	2–0.25	0.25–0.053
Enclosure land	72.77 ± 1.84 a	14.35 ± 2.77 c	1.69 ± 0.32 a
Prescribed-burning land	54.95 ± 3.59 b	32.38 ± 2.06 a	5.14 ± 1.47 a
Fuel-wood shrubland	69.66 ± 1.03 a	16.56 ± 0.98 c	2.05 ± 0.17 a
Pasture field	62.20 ± 3.74 ab	22.65 ± 2.00 b	4.16 ± 2.08 a
Maize field	53.94 ± 6.62 b	30.29 ± 5.24 ab	6.12 ± 2.35 a

Values are mean ± standard error. Values with different letters in a column indicate significant differences (analysis of variance; *P* < 5%) within the same aggregate.

**Table 2:** Two-way ANOVA for effects of land use and aggregate size on SOC, TN concentration and stock, MBC and C_mic_: C_org_ ratio within soil aggregates.

	Land use		Aggregate size		Land use × Aggregate size	
	*F*	*P*	*F*	*P*	*F*	*P*
Aggregate-associated SOC concentration	20.95	<0.001	9.25	0.001	0.32	0.952
Aggregate-associated TN concentration	18.25	<0.001	6.02	0.006	0.10	0.999
Aggregate-associated SOC stock	10.44	<0.001	337.91	<0.001	12.67	<0.001
Aggregate-associated TN stock	7.11	<0.001	433.68	<0.001	12.30	<0.001
Aggregate-associated MBC	10.00	<0.001	9.22	0.001	1.06	0.416
Aggregate-associated C_mic_: C_org_ ratio	5.97	0.001	4.66	0.017	2.04	0.075

**Table 3:** Principal components and component loadings extracted from the variables.

Variables	Principal components (PCs)	
	1	2
Large macro-aggregate	−0.954	0.126
Small macro-aggregate	0.934	−0.054
Micro-aggregate	0.894	−0.232
SOC	−0.213	0.945
TN	−0.194	0.948
MBC	0.295	0.922
C_mic_: C_org_ ratio	0.736	0.187

**Table 4:** Soil properties under different land uses.

Type	Available N (mg kg^−1^)	Available P (mg kg^−1^)	Available K (mg kg^−1^)	pH	Mechanical composition (%)		
					Clay (%)	Silt (%)	Sand (%)
Enclosure land	643.61 ± 64.02	6.74 ± 1.25	60.35 ± 8.07	8.21 ± 0.07	29.66 ± 3.42	26.07 ± 0.42	44.27 ± 3.02
Prescribed-burning land	630.97 ± 38.41	7.23 ± 0.97	53.92 ± 9.57	8.33 ± 0.06	30.83 ± 0.65	22.14 ± 2.78	47.04 ± 3.10
Fuel-wood shrubland	721.39 ± 38.74	8.16 ± 1.02	47.47 ± 6.64	8.28 ± 0.02	27.64 ± 2.11	21.53 ± 0.87	50.83 ± 2.91
Pasture field	503.61 ± 5.14	11.32 ± 0.51	63.97 ± 4.36	8.08 ± 0.03	27.03 ± 6.78	33.12 ± 3.74	39.84 ± 3.06
Maize field	645.75 ± 79.45	14.32 ± 7.17	76.18 ± 6.45	8.22 ± 0.18	36.15 ± 5.31	30.14 ± 1.68	33.71 ± 5.25

Values are mean ± standard error.
